# A universal UHPLC-CAD platform for the quantification of polysaccharide antigens

**DOI:** 10.1038/s41598-023-37832-4

**Published:** 2023-06-30

**Authors:** A. Corrado, M. De Martino, V. Bordoni, S. Giannini, F. Rech, S. Cianetti, F. Berti, C. Magagnoli, R. De Ricco

**Affiliations:** Technical R&D, GSK Via Fiorentina, 1, Siena, Italy

**Keywords:** Glycoconjugates, Polysaccharides

## Abstract

Several glycoconjugate-based vaccines against bacterial infections have been developed and licensed for human use. Polysaccharide (PS) analysis and characterization is therefore critical to profile the composition of polysaccharide-based vaccines. For PS content quantification, the majority of Ultra High Performance Liquid Chromatography (UHPLC) methods rely on the detection of selective monosaccharides constituting the PS repeating unit, therefore requiring chemical cleavage and tailored development: only a few methods directly quantify the intact PS. The introduction of charged aerosol detector (CAD) technology has improved the response of polysaccharide analytes, offering greater sensitivity than other detector sources (e.g., ELSD). Herein, we report the development of a universal UHPLC-CAD method (UniQS) for the quantification and quality evaluation of polysaccharide antigens (e.g., *Streptococcus Pneumoniae*, *Neisseria meningitidis* and *Staphylococcus aureus*). This work laid the foundation for a universal UHPLC-CAD format that could play an important role in future vaccine research and development helping to reduce time, efforts, and costs.

## Introduction

Development and advancement in the field of vaccine research has brought huge improvements to the quality of human life, contributing to the prevention of bacterial and viral infections^1^. For this purpose, several polysaccharide vaccines as well as glycoconjugate vaccines, composed of a polysaccharide or oligosaccharide antigen linked to a carrier protein, have been developed and commercialized^2–4^.

Nevertheless, continued research and efforts towards the discovery, improvement and coverage increase of new or already existing vaccines against invasive infections worldwide (e.g., *Streptococcus pneumoniae—*Spn, *Neisseria meningitidis—*Nm*, Staphylococcus aureus*—Sa, etc.) must be a priority, based on their continued impact on human safety^5^.

The repeating unit composition of Spn, Nm and Sa PS used for this work is reported in Table 1^6^.

**Table 1:** Repeating unit composition of Spn, Nm and Sa PS evaluated for a universal chromatography development.

Polysaccharide	Repeat unit^16^
**Neisseria meningitidis (Nm)**	
Group A	→ 6)-α-D-Man*p*NAc(3/4OAc)-(1 → OPO_3_ →
**Streptococcus pneumoniae (Spn)**	
Type 2	→ 3)-[α-D-Glc*p*A-(1 → 6)-α-D-Glc*p*-(1 → 2)]-α-L-Rha*p*-(1 → 3)-α-L-Rha*p*-(1 → 3)-c-L-Rha*p*-(1 → 4)-β-D-Glc*p*-(1 →
Type 3	→ 3)-β-D-Glc*p*A-(1 → 4)-β-D-Glc*p*-(1 →
Type 6A	→ 3)-α-L-Rha*p*-(1 → 3)-D-Rib-ol-(5 → P → 2)-α-D-Gal*p*-(1 → 3)-α-D-Glc*p*-(1 →
Type 8	→ 4)-α-D-Glc*p*-(1 → 4)-α-D-Gal*p*-(1 → 4)-β-D-Glc*p*A-(1 → 4)-β-D-Glc*p*-(1 →
Type 9N	→ 4)-α-D-Glc*p*NAc-(1 → 4)-α-D-Glc*p*A-(1 → 3)-α-D-Glc*p*-(1 → 3)-β-D-Man*p*NAc-(1 → 4)-β-D-Glc*p*-(1 →
Type 11A	→ 6)-[Gro-(1 → P → 4)]-α-D-Glc*p*2_0.6_/3_0.5_Ac_2_-(1 → 4)-α-D-Gal*p*-(1 → 3)-β-D-Gal*p*4_0.5_/6_0.5_Ac_2_-(1 → 4)-β-D-Glc*p*-(1 →
Type 12F	→ 4)-[α-D-Glc*p*-(1 → 2)-α-D-Glc*p*-(1 → 3)]-β-D-Man*p*NAcA-(1 → 4)-[α-D-Gal*p*-(1 → 3)]-α-L-Fuc*p*NAc-(1 → 3)-β-D-Gal*p*NAc-(1 →
Type 15B	→ 6)-[α-D-Gal*p*2_0.06_/3_0.12_/4_0.12_/6_0.55_Ac_4_-(1 → 2)-[Gro_0.7_-(2 → P → 3)]-β-D-Gal*p*-(1 → 4)]-β-D-Glc*p*NAc-(1 → 3)-β-D-Gal*p*-(1 → 4)-β-D-Glc*p*-(1 →
Type 17F	→ 3)-α-D-Gal*p*-(1 → 3)-[α-D-Gal*p*-(1 → 4)]-β-L-Rha*p*2Ac-(1 → 4)-α-L-Rha*p*-(1 → 2)-D-Ara-ol-(1 → P → 3)-β-L-Rha*p*-(1 → 4)-β-D-Glc*p*-(1 →
Type 19A	→ 3)-α-L-Rha*p*-(1 → P → 4)-β-D-Man*p*NAc-(1 → 4)-α-D-Glc*p*-(1 →
Type 20B	→ 3)-[α-D-Glc*p*-(1 → 6)]-[β-D-Gal*f*2Ac_0.9_-(1 → 4)]-α-D-Glc*p*NAc-(1 → P → 6)-α-D-Glc*p*-(1 → 6)-β-D-Glc*p*-(1 → 3)-β-D-Gal*f*5_0.9_/6_0.9_Ac_2_-(1 → 3)-β-D-Glc*p*-(1 →
Type 22F	→ 3)-α-D-Gal*f*-(1 → 2)-α-L-Rha*p*-(1 → 4)-β-D-Glc*p*A-(1 → 4)-[α-D-Glc*p*-(1 → 3)]-β-L-Rha*p*2Ac_0.8_-(1 → 4)-α-D-Glc*p*-(1 →
Type 33F	→ 5)-β-D-Gal*f*2Ac_0.5_-(1 → 3)-β-D-Gal*p*-(1 → 3)-[α-D-Gal*p*-(1 → 2)]-α-D-Gal*p*-(1 → 3)-β-D-Gal*f*-(1 → 3)-β-D-Glc*p*-(1 →
**Staphylococcus aureus (Sa)**	
Type 5	→ 4)-β-D-Man*p*NAcA-(1 → 4)-α-L-Fuc*p*NAc(**3OAc**)-(1 → 3)-β-D-Fuc*p*NAc-(1 →
Type 8	→ 3)-β-D-Man*p*NAcA(**4OAc**)-(1 → 3)-α-L-Fuc*p*NAc-(1 → 3)-α-D-Fuc*p*NAc-(1 →

Glcp, Glucose; Galp, Galactose; Galf, Galctofuramose; GlcpA, Glucuronic acid; GalpA, Galacturonic acid; GlcNAc, N-Acetyl Glucosamine; GalNAc, N-acetyl Galactosamin; Neu5Ac, N-Acetyl neuraminic acid (sialic acid); Rhap, Rhamnose; ManpN, N-Acteyl Mannosaminuronic acid; ManNAcA, N-Acetyl Mannuronic Acid; Ribf, Ribofuranose; GlcpNAc, N-Acetyl-Glucosamine; GalpNAc, N-Acetyl Galactosamine, ManpNAc, N-Acetyl Mannosamine; FucpNAc, N-Acetylfucosamine; PnepNAc, N-Actyl Pneumosamine FucNAc, N-Acetyl Fucosamine; Arabinitol, Ara-ol; Ribitol, Rib-ol; Gro, glycerol

Historically, the analysis of complex glycans has been challenging due to their diversity and chemical heterogenicity^7^. Traditional and robust analytical techniques for the analysis of carbohydrates, such as Gas Chromatography coupled with Mass Spectrometer (GC–MS) and UHPLC, are used to analyze carbohydrate composition prior to the preparation of appropriate monosaccharide derivatives^8,9^. In addition, the analysis of underivatized monosaccharides is becoming more and more relevant. For this purpose, high performance anion exchange chromatography with pulsed amphoteric detection (HPAEC-PAD) has become a reliable tool for the direct analysis of monosaccharides (routinely used by worldwide Official Medicines Control Laboratories OMCLs)^10,11^. HPAEC-PAD is characterized by very high levels of precision, reproducibility, and specificity for saccharide quantification. On the other hand, long polysaccharide antigens are difficult to analyze without any major sample pre-treatment steps to either (i) purify the saccharide from impurities / matrix (e.g. Solid Phase Extraction treatment) and to (ii) cleave the glycosidic bonds to release the monosaccharide units^12,13^. In addition, a hydrolysis step is usually polysaccharide specific as it depends on the repeating unit structure, properties, and glycosidic linkage. It is also not possible to identify a single monosaccharide which is conserved between all polysaccharide antigens (neither across the Spn serotypes), leading to serotype specific hydrolysis and chromatography (as already observed for *N. meningitidis* serogroups A, C, W and Y^12,14,15^).

Recently, hydrophilic interaction liquid chromatography (HILIC) coupled to an evaporative light scattering detector (ELSD), mass spectrometer (MS), refractive index detector (RID) and a charged aerosol detector (CAD) was reported to improve the analysis of underivatized glycans^16–20^. In this context, a recent publication highlights CAD detectors as a means to detect and separate several monosaccharides simultaneously, without derivatization, using HILIC and size exclusion chromatography (SEC)^21^. Classical UHPLC is therefore emerging as an effective multipurpose analytical tool for Quality Control (QC) analysis of polysaccharide.

For these reasons, new and innovative physico-chemical approaches were explored to develop a universal platform to characterize current and future polysaccharide-based multivalent vaccine candidates.

With the aim of supporting Spn process development and the production of a novel multi-valent candidate vaccine (currently in Ph2 clinical development) based on an innovative technology referred to as MAPS (Multiple Antigen Presenting System)^22^, the use of serotype specific hydrolysis and chromatographic methods is not feasible.

In this manuscript, we report the development of a universal technique for carbohydrate/polysaccharide quantification, herein referred as UniQS (Universal Quantification of polySaccharides). The technique allows for the quantification of both purified and non-purified polysaccharides resulting from the different steps of the bacterial fermentation process. Moreover, this approach aims to offer an alternative to the detectors (e.g., ELSD and ESI MS) that are commonly used during the analytical characterization and purification of oligosaccharides and polysaccharides for basic research needs^23–26^.

The new technique consists of two UHPLC methods, reversed phase (RP) UHPLC and HILIC, (UniQS-1 and UniQS-2, respectively) coupled to a CAD detector for the analysis and characterization of underivatized and untouched polysaccharides. As a case example, it is herein described the application of the methods on Spn different PSs; Nm serogroup A PS and Sa serogroup 5 and 8. These methods are able to quantify and evaluate the quality and content of polysaccharide antigens throughout the development and the production steps of the vaccine candidate (from fermentation broth to purified steps). The development of this new technology may facilitate the support of multi-valent polysaccharide based vaccine candidates, including the new breakthrough technology known as MAPS (Multiple Antigen Presenting System)^22^.

## Materials and methods

### Materials and reagents

All solvents and buffer additives were of HPLC grade (Sigma Aldrich, VWR, and Carlo Erba suppliers).

Spn PS, *Neisseria meningitidis* serogroup A PS and *Staphylococcus aureus* serogroup 5 and 8 PS were provided by GlaxoSmithKline (GSK).

### Instrumentation

Waters Acquity *H*-Class (Waters Corporation, Milford, MA), or Vanquish Thermo Flex (Thermo Fisher Scientific, Waltham, MA) systems, equipped with a quaternary pump, sample manager, column compartment, photodiode array (PDA) and charged aerosol (CAD) (Thermo Fisher Corona) detectors were used. Both chromatographic systems were equipped with 6-port switching valves to quickly direct the flow to the waste or detector. Furthermore, active pre-heater for eluents temperature stabilization before entering the column was used. The UV absorbance was recorded at 210 nm and 260 nm. Either chromatography UniQS-1 or UniQS-2 conditions are listed in the dedicate paragraph below. All data were collected and analyzed with Empower or Chromeleon software.

### Separation of PS by RP-CAD [UniQS-1]

Separation was carried out using a Acquity Premier BEH C4 column (1.7 µm 2.1 × 50 mm) equipped with a column guard (PN: 186004623) supplied by Waters. The mobile phase of the optimized method consisted of (A) water with 0.1% Trifluoracetic acid (TFA); and (B) acetonitrile (ACN) with 0.1% TFA. The following gradient elution was used: 2% B at 0–5 min, 2–10% B at 5–10 min, 10–34% B at 10–20 min, 34–90% B at 20–21 min, 90% B at 21–22 min, 90–2% B at 22–23 min and 2% B at 23–30 min (see Table 2 for additional details). The flow rate was 0.3 mL/min. A column temperature of 50 °C and an injection volume of 10 µL was used for the analysis. The following parameters were used for the CAD detector: (i) evaporation temperature: 55 °C; (ii) data collection rate: 2 Hz; (iii) noise filter: 3.6 s; (iiii) gas regulation mode: analytical; and time flow events: 0 min detector flow off, 8 min detector flow on, and 18 min detector flow off.

**Table 2:** Chromatographic details on time/elution conditions for UniQS-1.

Time (min)	Eluent A % (Water + 0.1% TFA*)	Eluent B % (ACN** + 0.1% TFA*)
Initial	98.0	2.0
5.0	98.0	2.0
10.0	90.0	10.0
20.0	66.0	34.0
21.0	10.0	90.0
22.0	10.0	90.0
23.0	98.0	2.0
30.0	98.0	2.0

*TFA = Trifluoracetic acid.

**ACN = Acetonitrile.

Chromatography was performed on both instruments, Vanquish Thermo and Waters Acquity, demonstrating the equivalence of the two systems.

### Separation of PS by HILIC-CAD [UniQS-2]

Separation was carried out using a LUNA 3 µm HILIC 200 Å (100 × 2 mm) equipped with a column guard (PN: AJ0-8786) supplied by Phenomenex. The mobile phase of the optimized method consisted of (A) ammonium formate 100 mM pH 3.5; (B) MilliQ water and (C) acetonitrile. The following gradient elution was used: 10% A, 20% B and 70% C at 0–3 min; 10% A, 20–90% B and 70–0% C at 3–18 min; 10% A, 90% B and 0% C at 18–20 min; 10% A, 90–20% B and 0–70% C at 20–21 min; and, 10% A, 20% B and 70% C at 21–30 min (see Table 3 for additional details). The flow rate was 0.4 mL/min. A column temperature of 30 °C and an injection volume of 10 µL was used for the analysis. The following parameters were used for the CAD detector: (i) evaporation temperature: 55 °C; (ii) data collection rate: 2 Hz; (iii) noise filter: 3.6 s; (iiii) gas regulation mode: analytical; and valves events: 0 min 6_1 switch valve, 2 min 1_2 switch valve, and 17 min 6_1 switch valve. The effects of column temperature (50 °C and 30 °C), CAD evaporation temperature (65, 60, 55 °C), and buffer ammonium formate pH 3.5 (25 mM, 20 mM and 10 mM) on chromatographic separation were evaluated.

Chromatography was performed on both instruments, Vanquish Thermo and Waters Acquity, demonstrating the equivalence of the two systems (Table 3).

**Table 3:** Chromatographic details on time/elution conditions for UniQS-2.

Time (min)	Eluent A % (ammonium formate 100 mM pH 3.5*)	Eluent B % (water)	Eluent C % (ACN*)
initial	10.0	20.0	70.0
3.0	10.0	20.0	70.0
18.0	10.0	90.0	0.0
20.0	10.0	90.0	0.0
21.0	10.0	20.0	70.0
30.0	10.0	20.0	70.0

*ACN = Acetonitrile.

## Results

### Optimization of gradient method via RP-UHPLC-CAD (RP-CAD) [UniQS-1]

**Figure 1:**
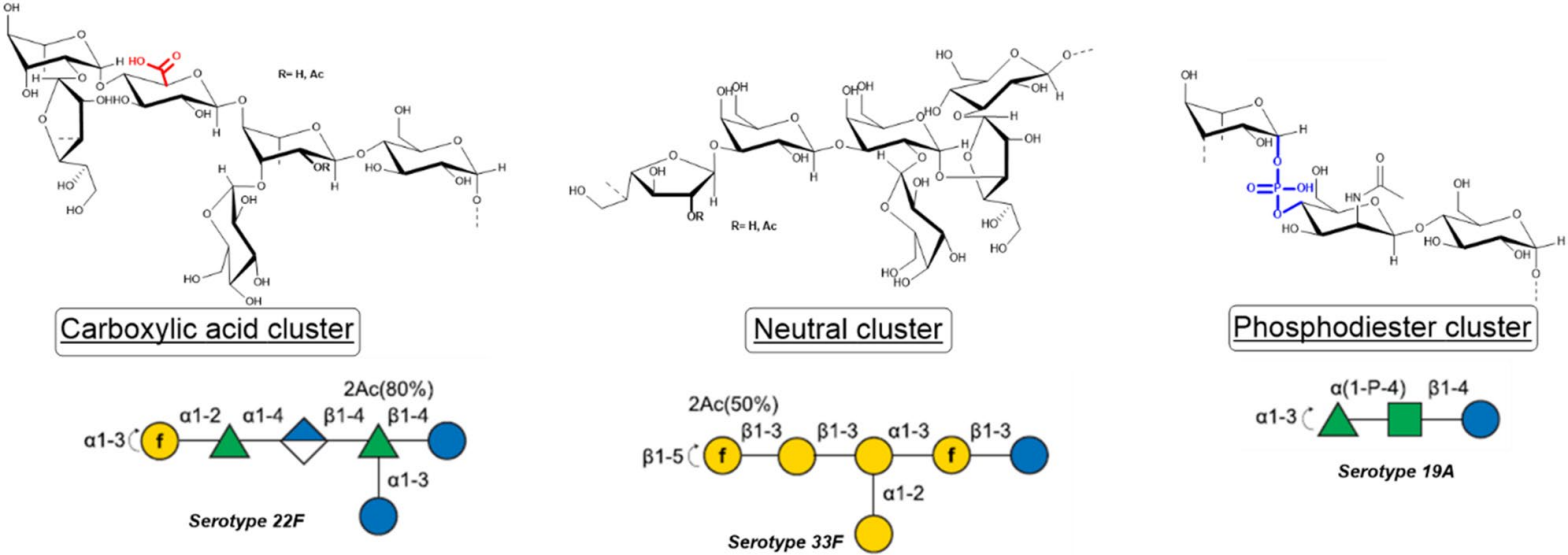
Serotypes cluster subdivision examples: carboxylic acid highlighted in red (left), phosphodiesters highlighted in blue (right), and neutral (middle).

Spn serotypes diverge by many characteristics: monosaccharide composition that defines the repeating unit, different anomeric linkages, different position linkages, and the presence of charges. Based on these characteristics, it was possible to identify three different clusters of serotypes. The polysaccharides were divided into neutral, carboxylic acid and phosphodiesters clusters (Fig. 1).

Overall, PSs show pronounced hydrophilic properties, due to their numerous functional groups (e.g. -OH, -COOH) and the presence of charged moieties. The backbone possesses hydrophobic regions, that could be exploited for the interaction with stationary phases in RP chromatography.

In the initial screening of conditions suitable for the retention of the polysaccharides, a classic RP mode by means of Water and ACN both added to 0.1% TFA was tested. A C4 based stationary phase with a large pore size (300 Å) was selected, considering the mass range of the PSs under evaluation (ACQUITY Premier Protein BEH C4 300 Å 1.7 µm). A low starting percentage of organic solvent was maintained (2%) to maximize PS interaction with the stationary phase. The initial runs demonstrated that the coupling of a CAD detector to the UHPLC systems was able to detect the polysaccharides peaks. The initial screening was performed on serotype 22F (clustered in the carboxylic acid group) by preparing a calibration standard curve based on the purified polysaccharide in water (concentrations ranging between 75 to 250 µg/mL).

**Figure 2:**
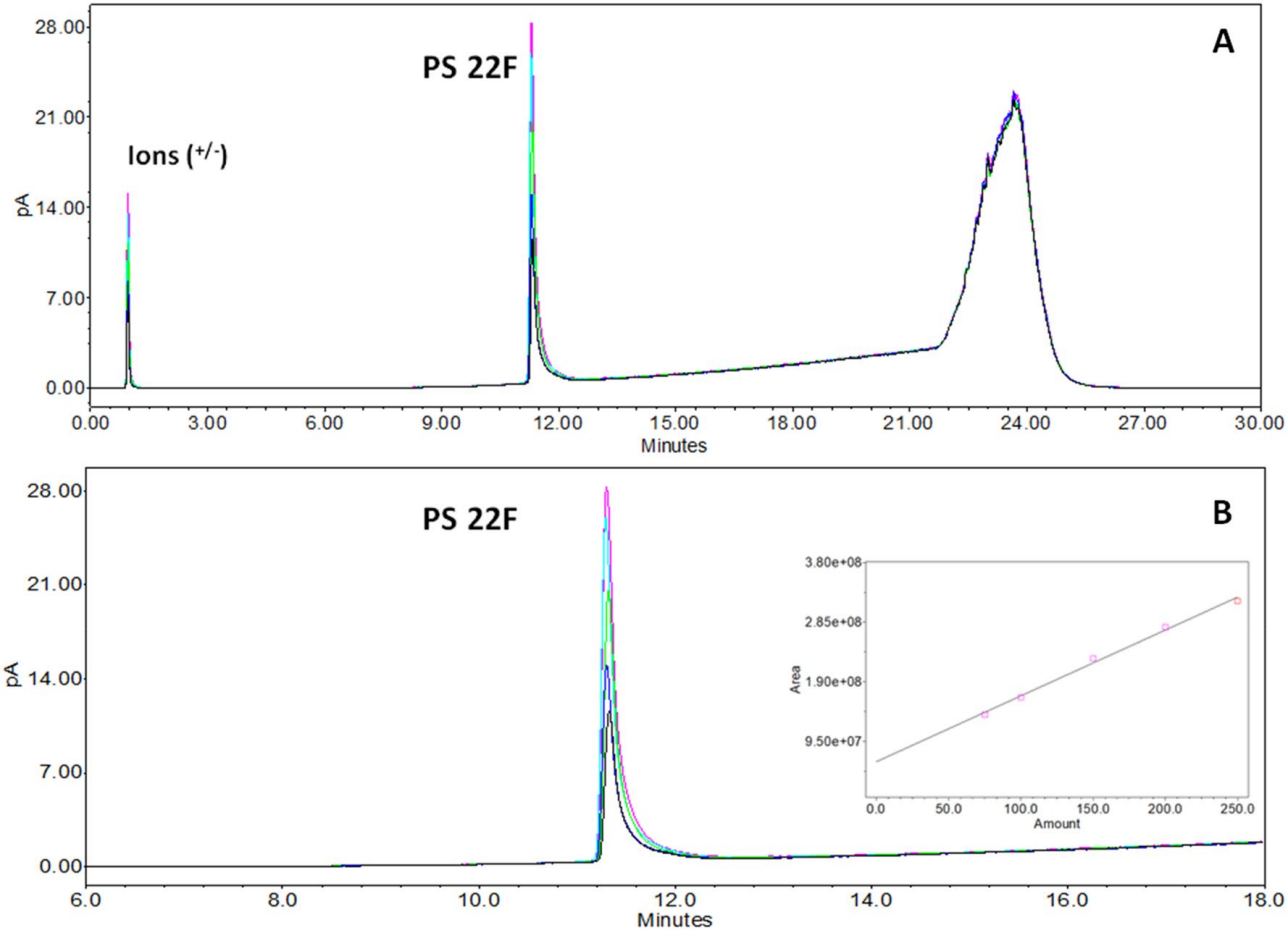
(**A**) Serotype 22F RP-CAD calibration curve linearity. The baseline rise observed between 22 and 25 min is also present in blank injections, and it is related to the change of mobile phase composition during the gradient elution. (**B**) Zoomed view (6 to 18 min) and calibration plot.

In RP-CAD mode by screening different eluting conditions using a C4-based stationary phase, the 22F serotype showed good detectability when the percentage of organic solvent was increased. Optimal elution conditions were obtained, resulting in a sharp and well-defined peak (a tailing effect, which is possibly linked to the polydispersity of the PS, is observed), by performing several DOEs to define the chromatographic conditions. As reported in Fig. 2, when building a calibration curve in the abovementioned range, the correlation between the theoretical concentration vs the measured area showed a good linearity (≥ 0.990) and the retention time remained unaltered between the different concentrations.

To expand the applicability to a wide range of matrices and to demonstrate the true universality of the developed chromatography method (UniQS-1), non-purified PSs, process intermediates of different PS purification steps (from the fermentation broth to the final purification steps) were tested (herein referred as Step 1 – fermentation broth to Step 4 – last purification step with cleaned PS).

**Figure 3:**
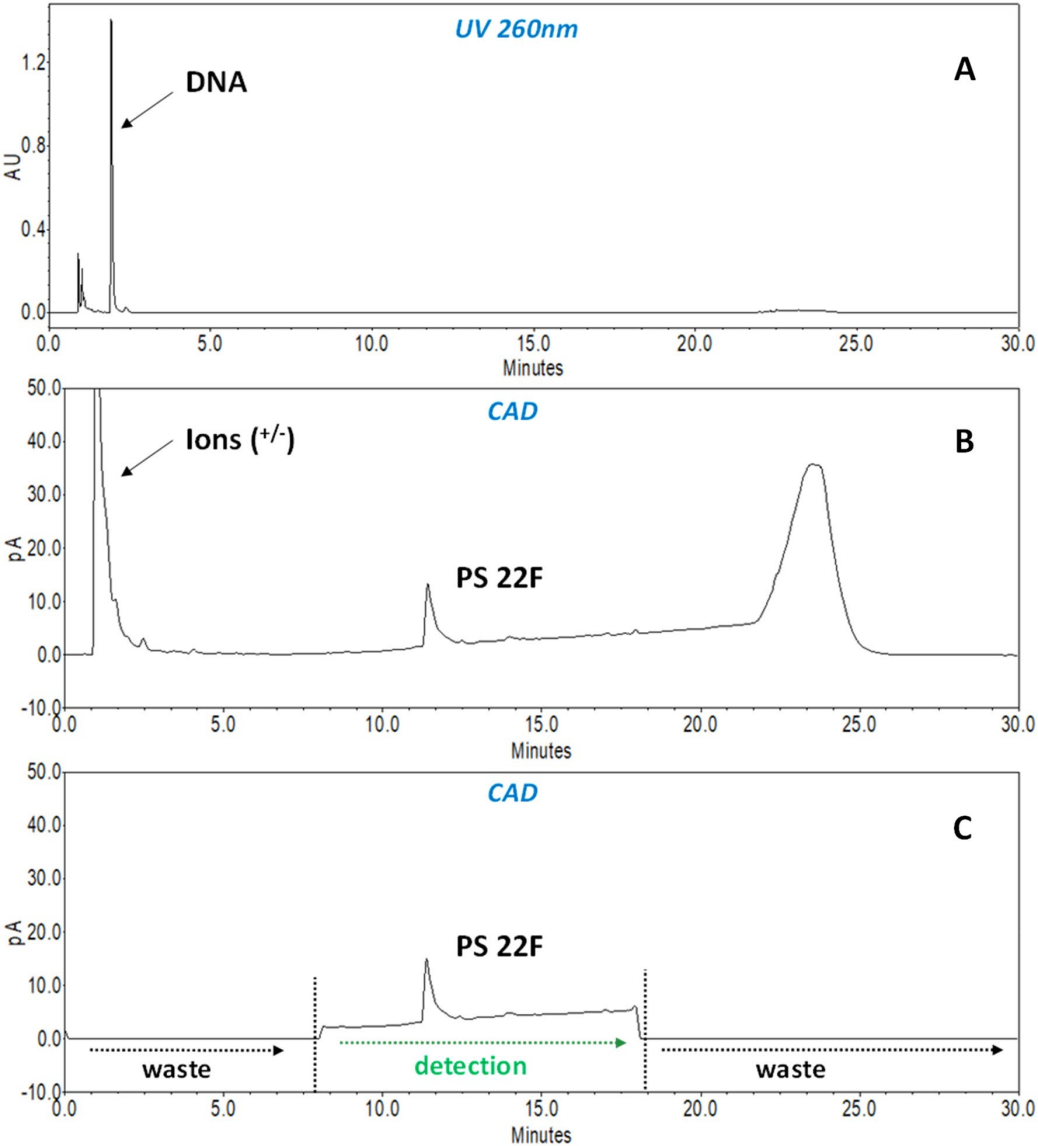
Comparison of Spn serotype 22F fermentation broth (step 1) with (**C**) and without events (**B**), acquired both with PDA (**A**) and CAD detectors (**B** and **C**). For details on instrumental flow state (to waste/to detector) setting, please refer to Materials and Method’s paragraph and Supplementary Fig. 4 for valves configuration).

To gather information around the elution of the many different compounds present in the fermentation broth (Step 1), the entire chromatographic run was acquired with both PDA and CAD detectors. As reported in Fig. 3A and B, the PS peak, which is easily detected at the same retention time as the purified standard, is completely separated from the process impurities (DNA, fermentation matrix, salts, surfactants, and others) providing a reliable quantification of the PS based on the purified standard. The applicability to the different purification steps gives the possibility to monitor the PS purification process step by step, accurately controlling and calculating PSs yields. It is to be noted that no sample pre-treatment was applied during method development.

To preserve detector functionality over time (due to the presence of salts and other abundant impurities), further refinements were introduced to the assay: the column outlet was connected to a 6-port valve that diverted the flow to waste at the beginning and end of the chromatographic run (where salts and other major contaminants are eluted), according to the standardized PSs elution window (Fig. 3C). In addition, to increase method robustness in terms of column shelf-life, the use of a guard column was implemented together with the introduction of simple pre-treatment steps (10 min centrifugation and 0,22 µm filtration) for process intermediates samples (Step 1 and following Steps before PS cleaning).

With these simple adjustments, the method applicability for routine testing in a quality control environment was increased significantly. Specificity to Cell-Wall Polysaccharide (CWPS), DNA, residual proteins and other contaminates was assessed in ad hoc studies, demonstrating the specificity of the assay to the serotype specific PS (CWPS showed a very broad and weak signal, which did not impact quantification; data shown in the supplementary information, Supplementary Fig. 1).

**Figure 4:**
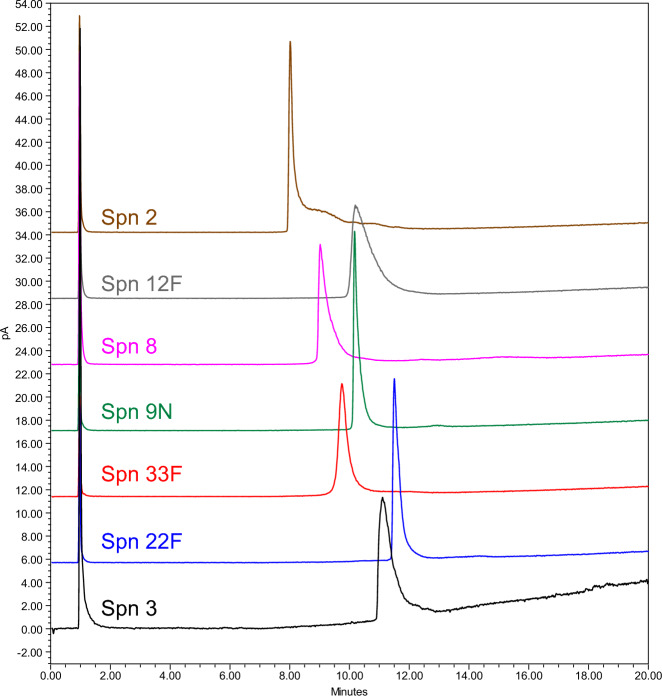
RP-CAD UniQS-1 profiles of Spn serotype 2, 12F, 8, 9N, 33F, 22F and 3 PSRP-CAD.

Following confirmation of the assay suitability to quantify the PS in all process phases (the purified PS was spiked in each phase and quantitative recovery was demonstrated – data not shown), the method was tested with other PS serotypes: 2, 3, 8, 9N and 12F. Each PS displayed linear behavior (Area vs Theoretical amount) over the standardized working range (75–250 µg/mL). Moreover, the retention time for each PS was confirmed for all tested PSs in a specific chromatographic window, although differences in area responses between serogroups were observed. (Fig. 4).

The method was subsequently tested against neutral polysaccharide clusters, starting from serogroup 33F. The neutral PS was found to elute in a defined window (8 to 14 min) and with an acceptable peak shape (Fig. 4). The linearity of the standard was also tested across different process purification steps, confirming the applicability of UniQS-1 to neutral PS clusters. As a further proof of the applicability of the assay to neutral PSs, Sa serotypes 5 and 8 PSs were tested with UniQS-1, both returned a standardized elution in the defined window (Supplementary Fig. 2).

**Figure 5:**
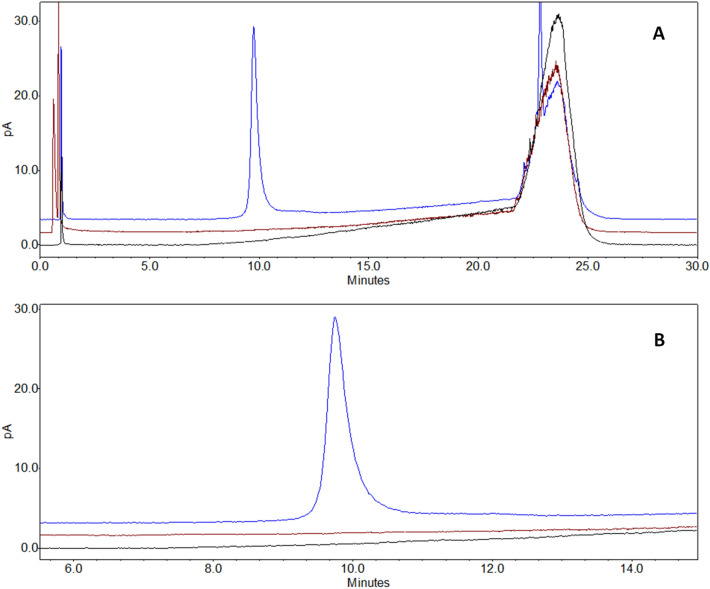
(**A**) RP-CAD UniQS-1 profiles of Spn Serotypes 11A (Black) and 19A (Brown) from phosphodiester cluster; 33F (Blue) from neutral cluster. (**B**) Zoomed view (6.0 to 14 min).

Finally, phosphodiester PS clusters were evaluated. Serotypes 11A and 19A (Fig. 5) were initially tested. As expected, due to the presence of phosphate groups (leading to a much higher polarity), these PSs were difficult to retain under the chromatographic conditions of the RP-CAD method. This phenomenon has also been reported for smaller analytes such as sugar phosphates, which are not retained in traditional RP chromatography; to improve the chromatographic retention of these compounds, a derivatization step is often proposed^27^. Other phosphodiester-based PS were tested in UniQS-1 with similar results, confirming that the assay is not a good option for this cluster.

### Development of a HILIC-CAD method for Spn serotype elution

Further screening of different stationary phases (C8 and Polyphenyl) confirmed that RP-CAD chromatography is not suitable to test PSs with phosphodiester groups. We therefore moved to evaluate alternative chromatographic approaches. Among them, Size Exclusion Chromatography was discarded as it demonstrated poor reproducibility (also due to the limitation of its applicability related to the solvents to be used in CAD detection). Finally, leveraging on a recent application published by Ghosh et al.^21^, the HILIC-CAD approach was explored.

**Figure 6:**
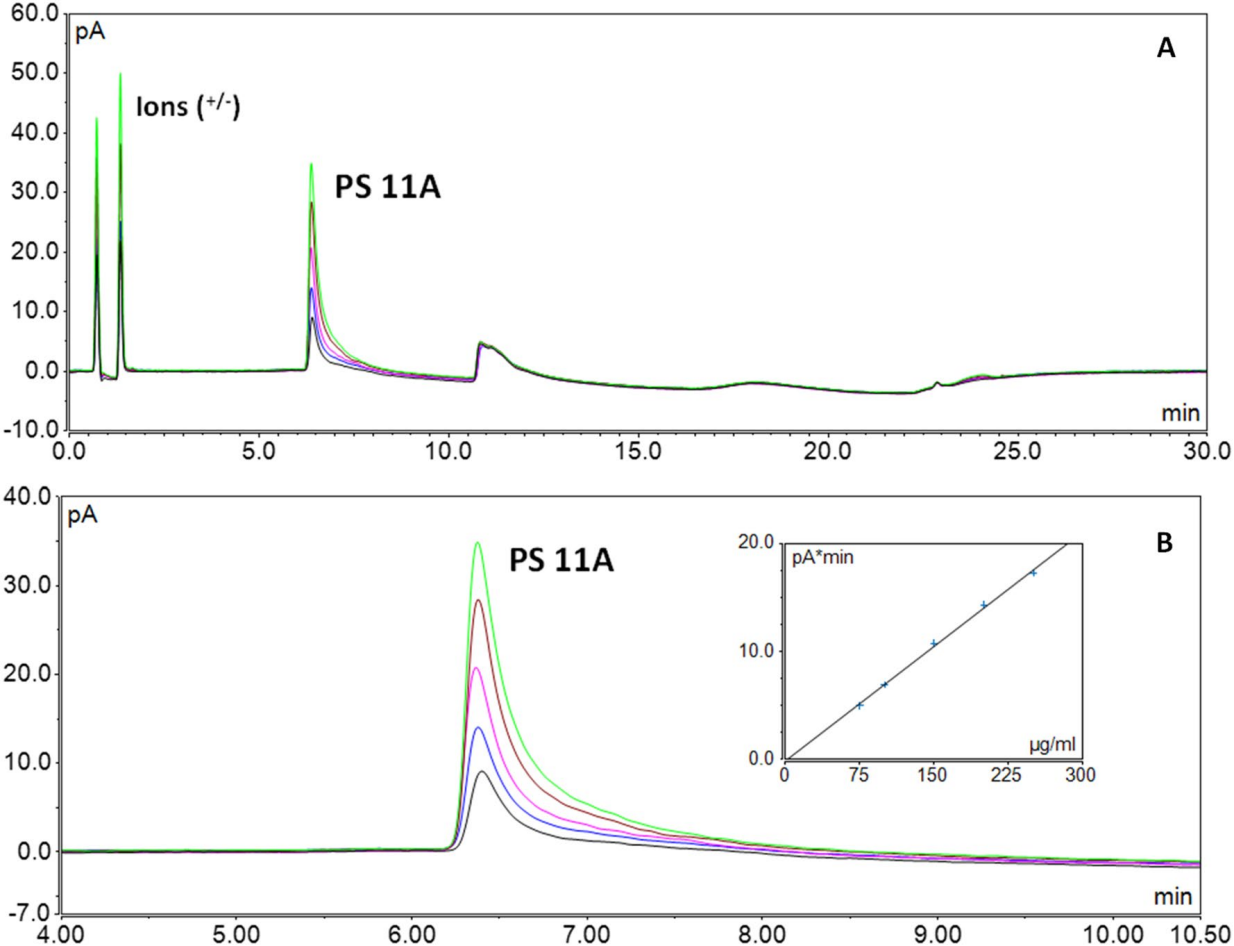
(**A**) HILC-CAD UNiQS-2 of Spn Serotypes. (**B**) Zoomed view (4 to 10.50 min) and calibration plot.

Development experiments were conducted (evaluating the type of gradient, buffer, and buffer concentration) to optimize the elution and detection of glycans containing a phosphodiesteric moiety within their structure. Hence, a combination of ammonium formate (10 mM pH 3.5), and acetonitrile was chosen for the optimized conditions. Moreover, sample preparation and concentration values for the calibration curve were kept consistent with the RP-CAD method. The serotype 11A was used as a case study for this cluster, and it showed good detectability for the selected analytical conditions, and good linearity over the range of standard concentrations for the calibration curve (Fig. 6).

**Figure 7:**
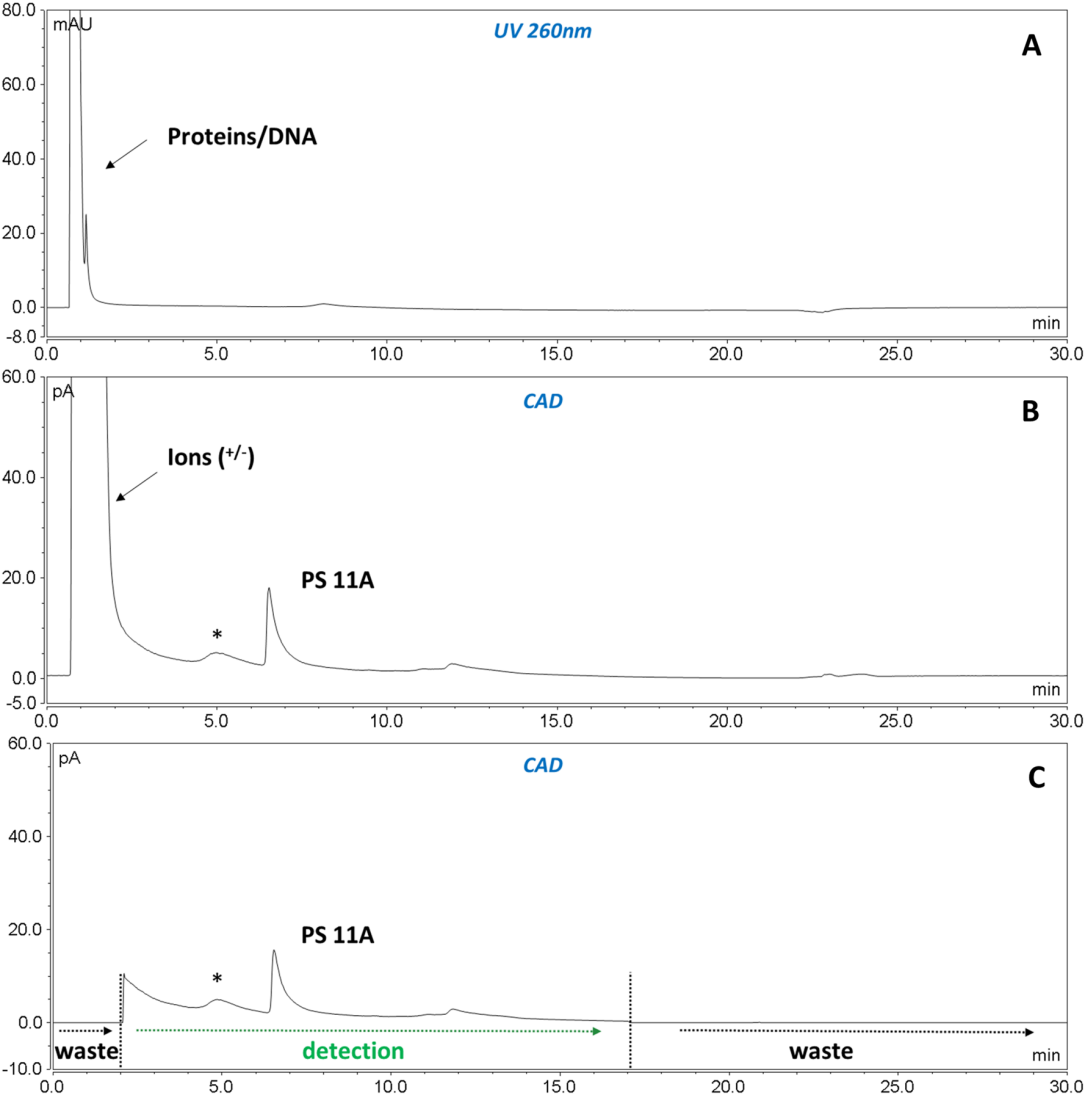
Comparison of serotype 11A fermentation broth (step 1) with (**C**) and without events (**B**), acquired both with PDA (**A**) and CAD detectors (**B** and **C**). * = PS related impurity, not present in purified PS (e.g. Step 4) as reported in Fig. 6.

As for UniQS-1, the applicability of the developed chromatography (UniQS-2) was extended to process intermediates (from the fermentation broth to the last purification steps) and several key process intermediates were tested (Fig. 7).

**Figure 8:**
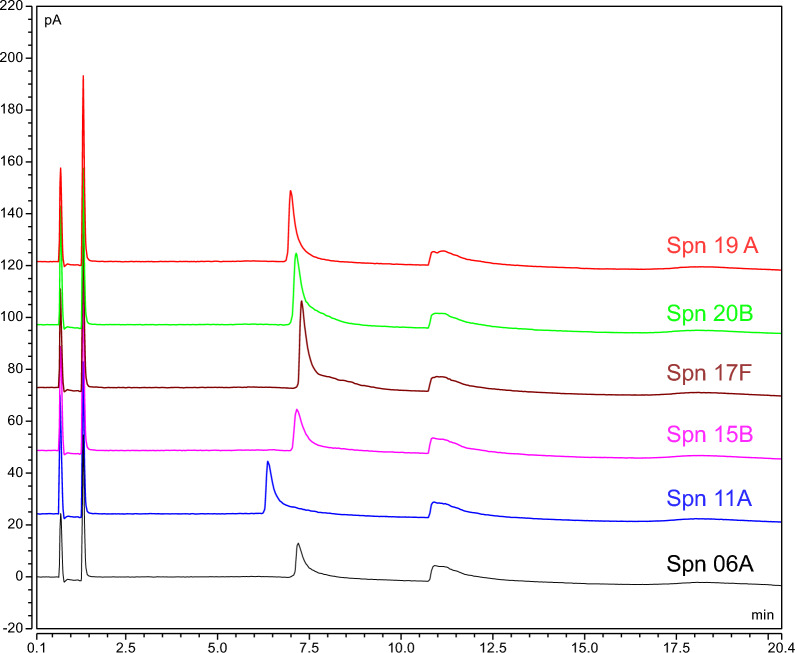
UniQS-2 profiles of Spn Serotypes, 19A, 20B, 17F, 15B, 11A, and 6A.

Additionally, each glycan serotype within the phosphodiester cluster group (6A, 10, 11A, 15B, 17F, and 20B) was injected and evaluated for method applicability on UniQS-2 (Fig. 8). The linearity of the curves of all tested polysaccharides confirmed the applicability of the method developed (UniQS-2). *Neisseria meningitidis* serogroup A PS was also tested with UniQS-2, confirming the applicability of this assay for polysaccharidic antigens of a different source (Supplementary Fig. 3). It is to be noted that, while Neutral PS (e.g. Spn serotype 33F or Sa serotype 5 and 8) are not eluted in UniQS-2, charged PS (carboxylic-acid cluster) can be eluted with a sharp peak in both UniQS-1 and UniQS2, expanding the chromatographic applicability to a wider range of substrates (data not shown).

## Discussion and conclusion

In this study, a universal UHPLC-CAD chromatography strategy (UniQS) for serotype cluster quantification and quality determination was developed to monitor polysaccharides during the production steps of (but not limited to) a pneumococcal vaccine candidate. To the best of our knowledge, this represents the first report of an underivatized PS chromatographic method for production monitoring of a multi-valent PS based vaccine. This UHPLC-CAD approach can monitor, detect, and quantify Spn PS serotype without sample derivatization or manipulation. Furthermore, samples are easily prepared for analysis by simple dilution to the target concentration. Good assay linearity was demonstrated for the quantification of polysaccharides; method specificity, precision and accuracy were also evaluated in light of future assay validation. The applicability to various PS purification steps was also demonstrated by testing at all stages of the production process, from the fermentation broth step to final PS purification steps, without any relevant sample pre-treatments. This simple and automatic UHPLC-CAD method has the potential to have an impact within the field of vaccines and biologics research and development because it can be applied to other substrates with similar characteristics. In addition, it could lead to material, economic and time savings in an industrial production process setting, supporting the development of ambitious multivalent vaccines that require a flexible analytical control strategy.

## Supplementary Information

Supplementary Information.

## Data Availability

The datasets used and/or analyzed during the current study available from the corresponding author on reasonable request.
