# Supplementary material for: A universal UHPLC-CAD platform for the quantification of polysaccharide antigens
Source: Sci Rep. 2023 Jun 30;13:10646. doi: 10.1038/s41598-023-37832-4 (PMC10313704; doi:10.1038/s41598-023-37832-4)
Supplement: Supplementary file 1 — Supplementary Information. [file 41598_2023_37832_MOESM1_ESM.docx]

**Supplementary information**

Supplementary Figure 1.UniQS-1 comparison of the CWPS profile (black), CPWS plus Spn serotype 22F (blue) and Spn serotype 22F (red). The elution region of CWPS is highlighted in the enlarged chromatogram.

Supplementary Figure 2. UniQS-1 profiles of Spn Serotypes, 22F; 33F and Sa serogroup type 5 and type 8


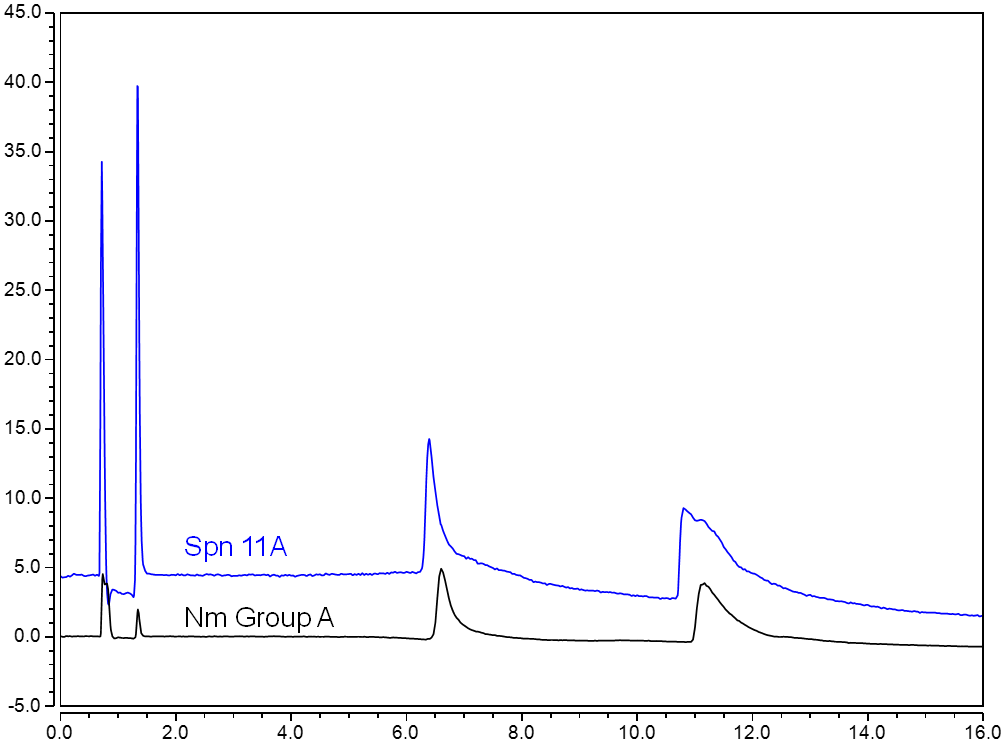


Supplementary Figure 3. UniQS-2 profiles of Spn Serotypes, 11A; 06A and Neisseria meningitidis group A


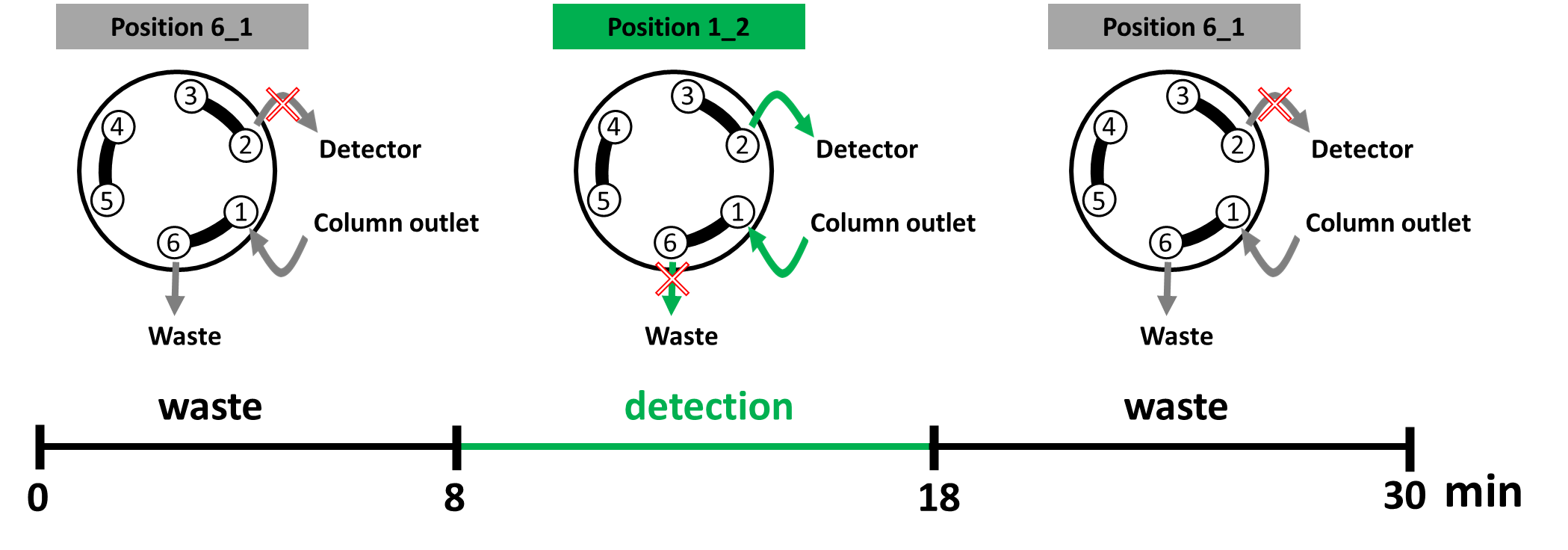


Supplementary Figure 4. UHPLC Valves configuration (to waste / to detector)
